# Supplementary material for: Antenna surface plasmon emission by inelastic tunneling
Source: Nat Commun. 2019 Oct 30;10:4949. doi: 10.1038/s41467-019-12866-3 (PMC6821910; doi:10.1038/s41467-019-12866-3)
Supplement: Supplementary file 1 — Supplementary information [file 41467_2019_12866_MOESM1_ESM.pdf]

Supplementary information for “Antenna surface plasmon emission by inelastic tunneling”

Zhang et al.

Supplementary note 1: Device fabrication

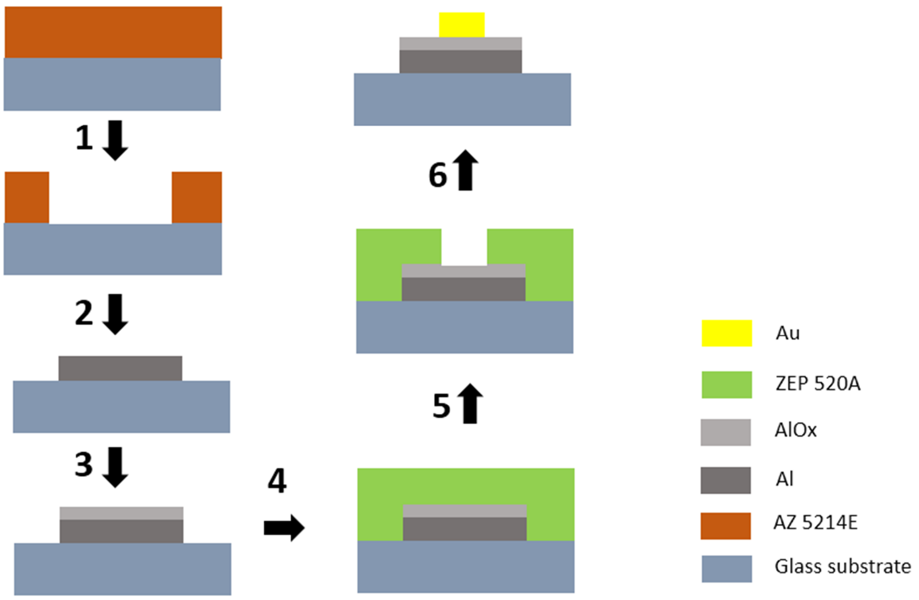

Supplementary Figure 1. Schematic illustration of the fabrication process. 1. Photolithography; 2. Al bottom electrode deposition; 3. Dry thermal oxidation; 4-5. Electron beam lithography; 6. Au top electrode deposition.

The fabrication of the Al/AlOx/Au junctions is based on two steps of lithography. For the substrate preparation, we use a standard silica substrate (VWR coverslip, 1.8cm × 2.2cm, the thickness is 0.15mm). Two 30-minutes ultrasonic bath cleaning processes are followed by acetone and IPA (Isopropyl Alcohol), respectively. Then, 10 minutes oxygen plasma (gas pressure 0.08 mBar and RF power 300W) is used to clean the substrate surface.

Photolithography is employed to define the Al bottom electrode. We spin-coat a layer of photoresist (AZ5214E) with a resist thickness of 1.4 µm. After prebaking on a hotplate at 110°C for 2 minutes, a single face mask aligner (SUSS Microtec MJB4) is used to facilitate the UV-exposure on the photoresist. The sample is then developed in AZ-400K solution (AZ-400K : water = 1:4) for 30s at room temperature, followed by rinsing with deionized water for 30s and drying in a stream of N_2. Next, an Al film with a thickness of 25nm is deposited via an e-beam evaporator (MEB 550, Plassys) under a vacuum pressure $< 5 \times 10^{-7}$ torr and an evaporation rate around 0.3nm/s. The sample is then

immersed in acetone at room temperature for 30 minutes to lift-off the photoresist. Finally, rinsing is done with IPA and drying with a N_2 flow.

To perform the dry thermal oxidation of aluminium, the sample is put inside a furnace at 200°C in atmosphere environment immediately after the lift-off. A 3nm of AlOx layer is obtained after three hours. The second lithography is electron-beam (e-Beam) writing using a NanoBeam nB4 lithography system. An e-beam resist (ZEP520A) with a thickness of 200nm and an electronic conductive layer (Espacer) are spin-coated successively. A prebaking is used at 170 °C for 3 minutes. After the e-beam exposure (with an e-beam voltage of 80kV, an e-beam current of 2 nA and a dose level of 4.8C/m^2), the development procedure includes: first, deionized water during 30 s to remove the conductive layer and rinsing in IPA for 30s; second, immersion of the sample in ZED-N50 for 90 s to develop the e-beam resist. Third, the sample is put in a mixed developper (MIBK:IPA=1:3) for 30s to complete the development of e-beam resist at a relatively low speed. Finally, the sample is rinsed in IPA for 30 s and dried with a stream of N_2. We then deposit 50 nm of Au (together with 1 nm of Cr as a stick layer) with the same e-beam evaporator using a deposition rate of 0.15 nm s^{-1}. The final lift-off is performed with Butanol for 3 hours.

The morphological qualities of the junction are assessed using SEM and AFM characterization. Supplementary figure 2 presents two SEM pictures showing the width variations along the antenna at two different positions. Width fluctuations (±10nm) can be seen. AFM measurement of the Al electrode (with 3nm AlOx layer) roughness are shown in supplementary figure 3. The root mean square value of the roughness is estimated to be below 1nm within $1\mu m^2$.

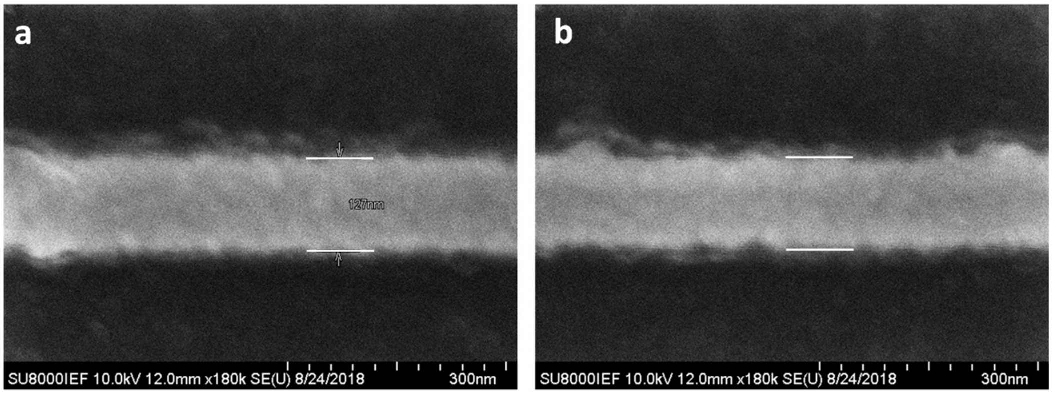

Supplementary Figure 2. SEM pictures showing the detailed shape of the patch antenna. (a) and (b) denote two different positions along one antenna wire. Two white lines (the distance between them is fixed at 127nm) indicate the edge of the patch wire. The scale bar is 300nm.

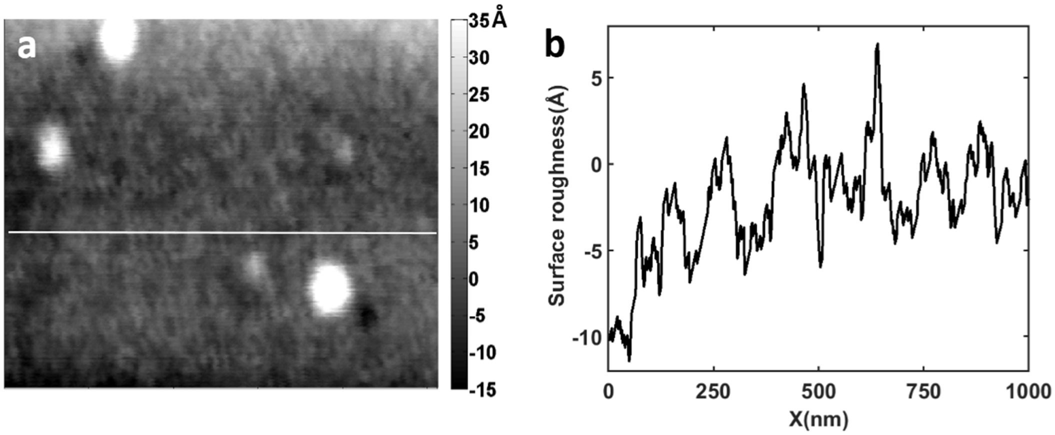

Supplementary Figure 3. AFM images of the AlOx layer profile. (a) Surface morphology of the AlOx layer in an area of 1µm×1µm; (b) A cross-section of the white line in figure (a) showing the surface roughness.

Supplementary note 2: Experimental setup for SPP emission characterization

The optical characterization for the Al/AlOx/Au junction is based on leakage radiation microscopy (LRM) using an inverted optical microscope (Olympus X71). We use an oil objective (NA=1.3, ×100) to collect the SPP emission through the glass substrate when the junctions are biased (positive bias on the Al electrode). The back focal plane imaging is schematically shown in supplementary figure 4. The real plane image can be captured by simply removing a lens. All the electroluminescence images are recorded with an electron multiplying CCD (EMCCD, Andor iXon 885) equipped with a water-cooling system. The integration time of the back focal plane and the real plane images of the

antenna junction are 900s with an EM gain of 3. For the planar junction without antenna, we use an integration time of 3900 s with an EM gain of 200. Regarding the spectral measurement, the generated light is injected into a multimode fiber by an objective (NA=0.5) and then guided into a spectrometer (Shamrock 750i). The integration time for acquiring the spectra in Fig. 3(a-c) is 520s, while the integration time for the spectra in Fig. 5(a) is 1040s.

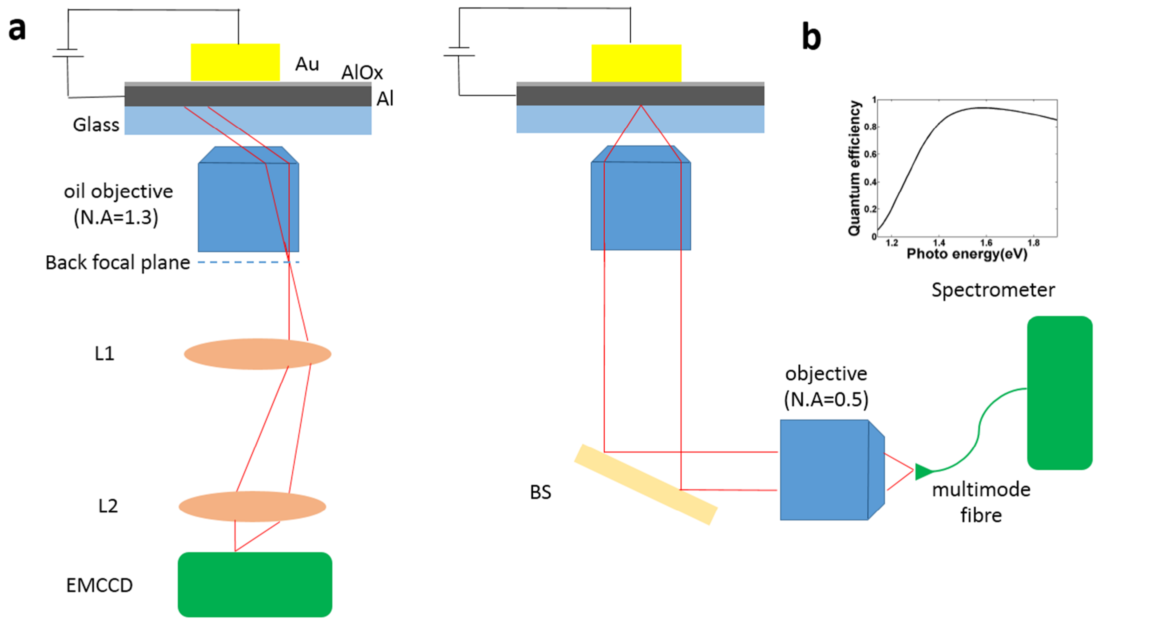

Supplementary Figure 4. Schematic of the optical characterization setup. (a) Back focal plane imaging, the orange lines indicate the optical path. (for real plane imaging, lens L2 is removed). (b) Experimental setup for spectrum measurement. The insert shows the quantum efficiency of the spectrometer.

Supplementary note 3: Time stability of the Al/AlOx/Au junction

Using a large-area Al/AlOx/Au tunnel junction with a high bias voltage at ambient temperature is difficult because of the electrical breakdown[1,2]. A poor heat dissipation efficiency decreases the lifetime of the junction. To avoid electrical breakdown at relatively high voltage, for instance at 1.6V, we used a pulsed voltage (1.5ms width and duty cycle of 50%) to bias the junction in order to let the system cool down. We have studied the electrical stability of the junction by recording the tunneling current during 10

minutes with a bias voltage of 1.6 V. As seen in supplementary figure 5, the current density is stable around a value of $5{\times}10^4$A/m^2 (except small fluctuations at some points), showing that the junction is electrically stable.

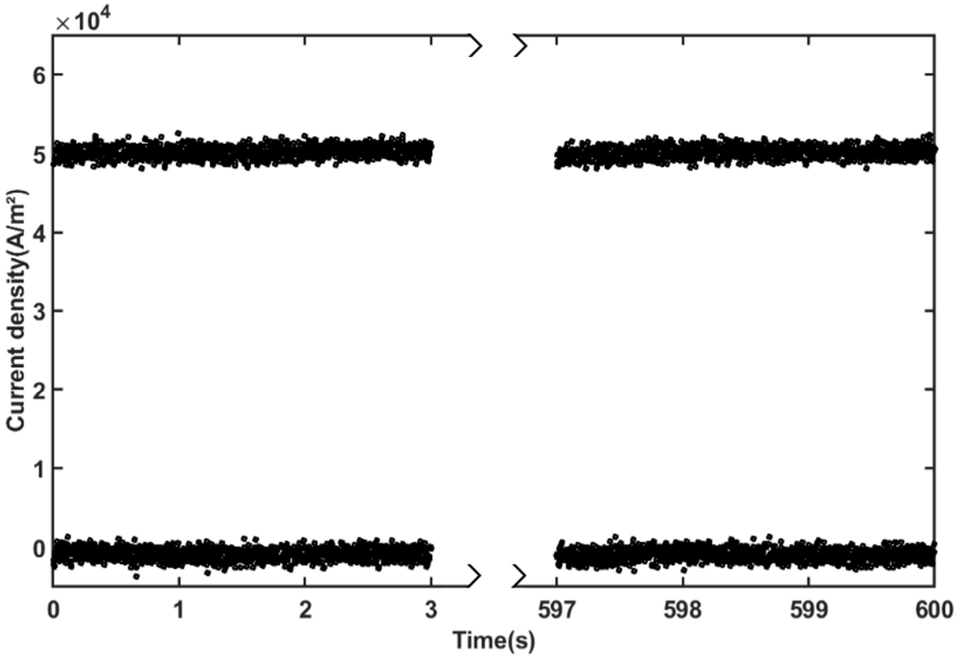

Supplementary Figure 5. Current stability of the tunneling current density (the same antenna junction as in figure 2) over 10 minutes when applying a square-shape pulsed voltage (peak value= 1.6V; base value=0V) with period 3 ms and duty cycle 50%.

Supplementary note 4: Additional *J*(*V*) characterization of the Al/AlOx/Au junction

In order to further check that the ASPEIT junction is in the tunneling regime, we measured the characteristic curve *J*(*V*) of the device under a range of temperature from 60K to 300K. We inserted the device in a crypto system (Cryostat OptiDry 150) and made five measurements. As shown in supplementary figure 6, it is clearly seen that the *J*(*V*) is weakly temperature dependent which confirms that the junction is in the tunneling regime.

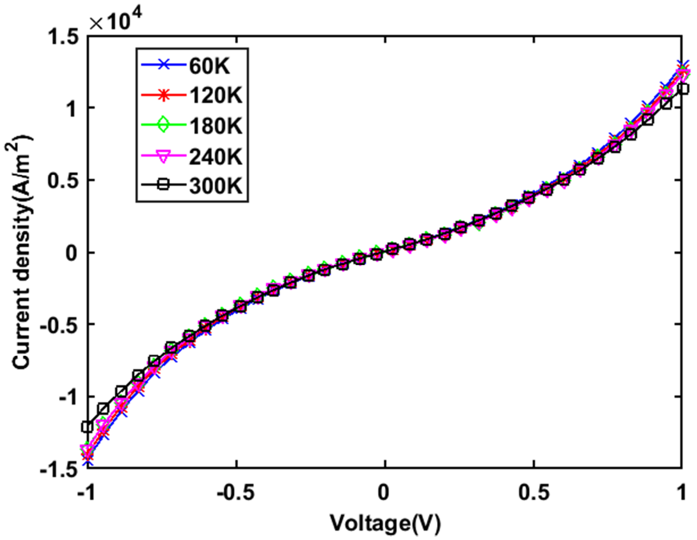

Supplementary Figure 6. *J*(V) characterization by varying the temperture. We recorded 5 *J*(V) curves in a temperature range varying from 60K to 300K with a step of 60K.

Moreover, we have studied the current as a function of the junction area. It is seen on supplementary figure 7 that the current increases linearly with the junction area from 7 μm^2 to 10^4 μm^2.

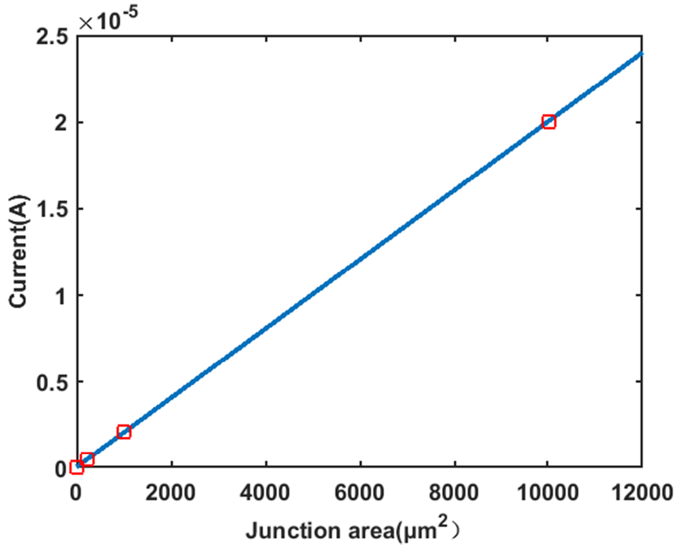

Supplementary Figure 7. Tunnel current vs junction area. We record 4 different junctions by varying the junction area from 7μm^2 to 10^4 μm^2 under the same bias voltage of 0.5V. The linear fit gives a guide to the eyes.

Supplementary note 5: Theoretical model of light emission by tunnel junction in presence of a nanoantenna

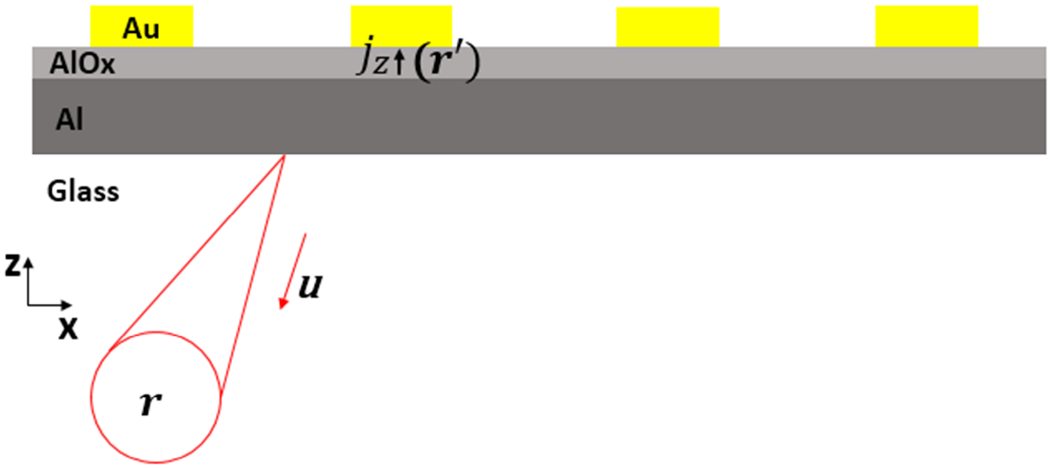

Supplementary Figure 8. Illustration of reciprocity principle for the light emission of a tunnel junction. **r** is a position in the far-field, **r**' is a current element position in the tunnel barrier.

Light emission from a tunnel junction can be modelled as the field radiated by the current density fluctuations of the tunneling current[3-5]. The power spectral density of the current density fluctuations is derived from a model of the tunneling current.[4,6,7] As shown in supplementary figure 8, the radiation through the substrate is due to the current fluctuation of the tunneling current density in the z-direction (j_z). The power dP radiated through the substrate in the direction **u** in a solid angle subtended by the area dA is given by:

$$dP = \left\langle \mathbf{E}(\mathbf{r},t) \times \mathbf{H}(\mathbf{r},t) \right\rangle \mathbf{u} \cdot dA, \tag{1}$$

where **E**, **H** are the electric and magnetic field, respectively. The brackets denote a statistical average over an ensemble of realizations of the fluctuating current. We now introduce the Fourier transform of the electromagnetic field and current density:

$$\begin{aligned} \mathbf{E}(\mathbf{r},t) &= \int \mathbf{E}(\mathbf{r},\omega) \cdot e^{-i\omega t} \frac{d\omega}{2\pi}, \\ \mathbf{H}(\mathbf{r},t) &= \int \mathbf{H}(\mathbf{r},\omega) \cdot e^{-i\omega t} \frac{d\omega}{2\pi}, \end{aligned} \tag{2}$$

$$j_z(\mathbf{r},t) = \int j_z(\mathbf{r},\omega) \cdot e^{-i\omega t} \frac{d\omega}{2\pi}. \tag{3}$$

For a stationary process, the Fourier transform does not exist in the sense of a function. However, the power spectral density $S_{j_n,j_m}(\mathbf{r},\mathbf{r}',\omega)$ can be defined and we can write:

$$\left\langle j_n(\mathbf{r},\omega) j_m(\mathbf{r}',\omega')\right\rangle = 2\pi\delta(\omega+\omega')\, S_{j_n,j_m}(\mathbf{r},\mathbf{r}',\omega). \tag{4}$$

Note that $j_m(\mathbf{r}',\omega') = j_m^*(\mathbf{r}',-\omega')$ as $j_m(\mathbf{r}',t)$ is a real function. The electric field at $\mathbf{r}$ radiated by the current density in the tunnel junction[8], is given by

$$\mathbf{E}(\mathbf{r},\omega) = i\omega\mu_0 \int d^3\mathbf{r}'\,\mathbf{G}(\mathbf{r},\mathbf{r}',\omega)\mathbf{j}(\mathbf{r}',\omega)\ , \tag{5}$$

where $\mathbf{G}$ is the Green tensor of the system. To specify the polarization, we introduce the unit vector $\mathbf{e}^l$ where l=s or p so that $\mathbf{E} = E^l\mathbf{e}^l + E^p\mathbf{e}^p$. Using Einstein notation, the n -th component of the l-polarized electric field amplitude is

$$E_n^l(\mathbf{r},\omega) = i\omega\mu_0 \int d^3\mathbf{r}'\, e_n^l G_{nm}(\mathbf{r},\mathbf{r}',\omega) j_m(\mathbf{r}',\omega)\ . \tag{6}$$

Based on (1), the radiated power in l-polarization is

$$dP^l = \int\frac{d\omega}{2\pi}\int\frac{d\omega'}{2\pi}\, e^{-i(\omega+\omega')t}\left\langle \mathbf{E}^l(\mathbf{r},\omega)\times\mathbf{H}^l(\mathbf{r},\omega')\right\rangle\cdot\mathbf{u}\ dA. \tag{7}$$

By using $\mathbf{H}^l(\mathbf{r},\omega) = \dfrac{\mathbf{B}^l(\mathbf{r},\omega)}{\mu_0}$ and $\mathbf{B}^l(\mathbf{r},\omega) = \dfrac{\mathbf{k}}{\omega}\times\mathbf{E}^l(\mathbf{r},\omega) = \dfrac{n}{c}\mathbf{u}\times\mathbf{E}^l(\mathbf{r},\omega)$ we get:

$$dP^l = n\varepsilon_0 c\int\frac{d\omega}{2\pi}\int\frac{d\omega'}{2\pi}\, e^{-i(\omega+\omega')t}\ \left\langle \mathbf{E}^l(\mathbf{r},\omega)\cdot\mathbf{E}^l(\mathbf{r},\omega')\right\rangle dA. \tag{8}$$

Using (4) and (6), we find:

$$dP^l = n\varepsilon_0 c\int\frac{d\omega}{2\pi}\mu_0^2\omega^2\int d^3\mathbf{r}'\, e_n^l G_{np}(\mathbf{r},\mathbf{r}',\omega)\int d^3\mathbf{r}''\, e_n^l G_{nq}^*(\mathbf{r},\mathbf{r}'',\omega)\, S_{jp,jq}(\mathbf{r}',\mathbf{r}'',\omega) dA. \tag{9}$$

Here, we only consider the z-component of the current density in the junction, so that p=q=z:

$$dP^l = n\mu_0 c\int\frac{d\omega}{2\pi}\left(\frac{\omega^2}{c^2}\right)\int d^3\mathbf{r}'\, e_n^l G_{nz}(\mathbf{r},\mathbf{r}',\omega)\int d^3\mathbf{r}''\, e_n^l G_{nz}^*(\mathbf{r},\mathbf{r}'',\omega)\, S_{jz,jz}(\mathbf{r}',\mathbf{r}'',\omega)\ dA. \tag{10}$$

To proceed, we use a simplified form of the two points correlation function:

$$S_{jz,jz}(\mathbf{r},\mathbf{r}',\omega) = \frac{\left\langle I^2\right\rangle(\omega)}{\Sigma}\delta(\mathbf{r}-\mathbf{r}'), \tag{11}$$

where $\left\langle I^2 \right\rangle(\omega)$ is the intensity power spectral density and $\sum$ represents the area of the junction. This approximation is based on the fact that the current density in different electronic modes are uncorrelated and that a typical mode occupies an area given by λ_F^2/π, where λ_F is the Fermi wavelength on the order of a few angstroms. We insert Eq. (11) into Eq. (10), and introduce the solid angle $dA = r^2 d\Omega$ and the vacuum impedance $Z_0 = \mu_0 c$. Moreover, we note that, since the gap is deeply subwavelength, the field does not vary rapidly along z in the gap so that the integration over z and z' yields a t^2 dependence where t is the barrier thickness of the tunnel junction. This assumption may not be correct[5,9], when using antennas as the fluctuating current in the antenna may couple efficiently to the plasmonic mode. This is expected to impact the amplitude of the emitted field but not its spectrum, polarization and angular pattern which are given by G. Thus we obtain:

$$dP^l = Z_0 n \int d\omega \left(\frac{t^2 \omega^2}{c^2} \right) \left\langle I^2 \right\rangle(\omega) \int \frac{d^2 \mathbf{r}_{\parallel}'}{\Sigma} r^2 \left| e_n^l G_{nz}(\mathbf{r}, \mathbf{r}_{\parallel}', 0, \omega) \right|^2 d\Omega, \tag{12}$$

where $\mathbf{r}_{\parallel}' = (x', y', 0)$. Now we show that the term $r^2 \left| G_{nz}(\mathbf{r}, \mathbf{r}_{\parallel}', 0, \omega) \right|^2$ can be related to the field enhancement factor in the junction by using the reciprocity theorem[8]. To proceed, we consider a dipole source at $\mathbf{r} = r\mathbf{u}$ in the far field with a dipole moment $p_{\mathrm{inc}} \cdot \mathbf{e}^l$. Using equation (6), we see that this dipole generates a field in the junction at $\mathbf{r}'$:

$$E_z^l(\mathbf{r}', \omega) = \omega^2 \mu_0 G_{zn}(\mathbf{r}', \mathbf{r}, \omega)\, p_{inc} e_n^l. \tag{13}$$

The same dipole in vacuum generates a field, whose amplitude is given by:

$$E_{inc} = \mu_0 \omega^2 \frac{e^{ikr}}{4\pi r} p_{inc}, \tag{14}$$

By taking the ratio of the field in the gap (s-13) and the incident field (s-14) illuminating the junction, we define the field enhancement factor:

$$K^l(\mathbf{r}', \mathbf{u}, \omega) = \left| \frac{E_z(\mathbf{r}', \mathbf{u}, \omega)}{E_{inc}(\mathbf{r}', \mathbf{u}, \omega)} \right| = 4\pi r \left| G_{zn}(\mathbf{r}', \mathbf{r}, \omega) e_n^l \right|. \tag{15}$$

The reciprocity theorem gives:

$$G_{zn}(\mathbf{r}', \mathbf{r}, \omega) e_n^l = e_n^l G_{nz}(\mathbf{r}, \mathbf{r}', \omega). \tag{16}$$

using (13), (14) and (16), we obtain:

$$K^l(\mathbf{r}',\mathbf{u},\omega)=\left|\frac{E_z}{E_{inc}}\right|=4\pi r\left|e_n^l G_{nz}(\mathbf{r},\mathbf{r}_{||}',0,\omega)\right|. \quad (17)$$

Inserting (17) into (12), we have

$$dP^l=Z_0 n\int\frac{d\omega}{2\pi}\left(\frac{\omega^2 t^2}{c^2}\right)\int\frac{d^2\mathbf{r}'}{\Sigma}\frac{\left|K^l(\mathbf{r}',\mathbf{u},\omega)\right|^2}{16\pi^2}\left\langle I^2\right\rangle(\omega)\ d\Omega. \quad (18).$$

Finally, the power can be cast in the form:

$$\frac{dP^l}{d\Omega}=Z_0 n\int\frac{d\omega}{8\pi}\left(\frac{t}{\lambda}\right)^2\overline{\left|K^l(\mathbf{u},\omega)\right|^2}\left\langle I^2\right\rangle(\omega), \quad (19)$$

where we take $\overline{\left|K^l(\mathbf{u},\omega)\right|^2}$ to be the averaged field enhancement factor over the whole junction barrier. We now introduce the differential radiation impedance ($R^l(\mathbf{u},\omega)$) of a tunnel junction with unit $\Omega\, sr^{-1}$, which accounts for the coupling between the tunneling current and the power spectral density of the power radiated per solid angle. Based on this, (19) can be written as:

$$\frac{dP^l}{d\Omega}=\int d\omega\ R^l(\mathbf{u},\omega)\ \left\langle I^2\right\rangle(\omega), \quad (20)$$

where $R^l(\mathbf{u},\omega)=\frac{Z_0 n}{8\pi}\left(\frac{t}{\lambda}\right)^2\overline{\left|K^l(\mathbf{u},\omega)\right|^2}$. The physical content of this expression is very simple. We remind that a dipolar wire with length t and monochromatic current with intensity I radiates a power $RI^2=\frac{\pi}{3}Z_0\left(\frac{t}{\lambda}\right)^2 I^2$. It follows that the junction behaves as an emitting electric dipole. The key difference is the enhancement factor provided by the antenna. This enhancement factor becomes significant if the plasmonic mode is excited, namely for a specific angle of incidence and for specific frequencies.

We now simplify the expression of the power emitted by taking into account the fact that the antenna selects a bandwidth centered at a particular frequency. If we consider a specific bandwidth with a central frequency of ω_0, which can correspond to a resonant antenna, it can be cast as:

$$\frac{dP^l}{d\Omega} = R^l(\mathbf{u},\omega_0)\left\langle I^2\right\rangle(\omega_0)\int d\omega \; \frac{R^l(\mathbf{u},\omega)}{R^l(\mathbf{u},\omega_0)} \frac{\left\langle I^2\right\rangle(\omega)}{\left\langle I^2\right\rangle(\omega_0)}, \qquad (21)$$

$$= R^l(\mathbf{u},\omega_0)\left\langle I^2\right\rangle(\omega_0)\;\Delta\omega$$

where $\Delta\omega = \int d\omega \; \frac{R^l(\mathbf{u},\omega)}{R^l(\mathbf{u},\omega_0)} \frac{\left\langle I^2\right\rangle(\omega)}{\left\langle I^2\right\rangle(\omega_0)}$ denotes the bandwidth of the antenna mode.

For a standard junction, our implementation of current fluctuation is based on the model from the reference[10]:

$$\left\langle I^2\right\rangle(\omega) = \frac{1}{1-\exp(-\frac{eV}{k_B T}(1-\frac{\hbar\omega}{eV}))} \; eI_0(1-\frac{\hbar\omega}{eV}), \qquad (22)$$

where I_0 is the tunneling current, e is the electron charge, ω is the frequency, $\hbar$ is the reduced Planck's constant, k_B is Boltzmann's constant, and T is temperature. Moreover, considering a non-linearity for the I-V characteristic of a tunnel junction, it is important to point out the current fluctuation should behave like[3,6]:

$$\left\langle I^2\right\rangle(\omega) = \frac{1}{1-\exp(\frac{eV}{k_B T}(1-\frac{\hbar\omega}{eV}))} \; eI(V-\frac{\hbar\omega}{e}), \qquad (23)$$

where $I(V)$ is the dc characteristic of the voltage-biased tunnel junction. Here, we have neglected the term proportional to $I(V+\frac{\hbar\omega}{e})$ because the weighting factor $\frac{1}{\exp\{(eV+\hbar\omega)/k_B T\}+1} \ll 1$. We should emphasize that the absolute value of the current fluctuation from the (22) is larger than the (23).

In terms of the electron-to-photon conversion efficiency, based on (21), we approximately write the efficiency:

$$\eta_{e-p} = \frac{N_{photon}}{N_{electron}} = \frac{dP^l}{\hbar\omega_0} \; \frac{e}{I(V)} = 2\pi \frac{R^l(\mathbf{u},\omega_0)\left\langle I^2\right\rangle(\omega_0)\;\Delta\omega\;\Delta\Omega}{\hbar\omega_0} \; \frac{e}{I_0}. \qquad (24)$$

Introducing the quantum of resistance $R_k = h/e^2$, we have:

$$\eta_{e-p}(\omega_0) = \left[\frac{Z_0}{R_k}\right] \frac{\Delta\omega}{\omega_0} \; \frac{n}{4}\left(\frac{t}{\lambda_0}\right)^2 \overline{\left|K^l(\mathbf{u},\omega_0)\right|^2} \; \frac{\left\langle I^2\right\rangle(\omega_0)}{eI_0}\Delta\Omega. \qquad (25)$$

Note that in the case of a planar junction, the "enhancement" $|K|^2$ is smaller than 1 as the incident field decays when propagating through the metals before reaching the junction barrier. For a 25 nm thick aluminium layer, $|K|^2$ is on the order of 5×10^{-3}. Conversely, when using resonant antennas, this factor of $|K|^2$ is on the order of 70. Note the value of $|K|^2$ is taken from a specific emission angle where corresponds to the plasmon emission (at air/Al interface). In a word, we can see that the enhanced efficiency is determined by the field enhancement factor in the tunnel barrier.

To implement this model, we need to compute the field enhancement in the gap numerically. We use the aperiodic-Fourier modal method[11]. We consider the reciprocal situation and illuminate the ASPEIT junction by a plane wave through the substrate side. We consider one fixed position in the middle of the tunnel barrier (we assume the field along the z-direction of AlOx layer is homogeneous). In order to compute the power emitted in a particular direction, we need to compute the corresponding enhancement factor. To proceed, we illuminate with a plane wave coming from this direction specified by a polar angle varying from 0º to 60º (with 70 points, which corresponds to the numerical aperture of the objective), and an azimuthal angle varying from 0º to 360º (with 200 points). Furthermore, we average the intensity enhancement factor over the width of the antenna using 15 points. In order to compute an emission spectrum, we repeat the simulation for 100 different frequencies. To account for antenna width fluctuations, we average 7 spectra from 7 different widths varying from 116nm to 140nm. To reduce time consumption for the calculation, we scan one-quarter of the azimuthal angles taking advantage of the symmetry of the emission diagram. A single frequency computation with a computer equipped with 16 cores and 100GB memory, takes nearly 20 minutes.

The model allows investigating the electron-to-photon conversion efficiency. The ASPEIT junction has a theoretical efficiency of 1.1×10^{-7}, while the planar junction, has an estimated efficiency of 2.7×10^{-10}. It is important to note that the theoretical prediction of the ASPEIT junction is one order of magnitude lower than the experiment result. Similar issue has been mentioned for calculating the photon emission from a planar junction[5]. As mentioned above, we attribute this discrepancy to the simplified form of the current density correlation function. Since the thickness of Au top electrode is close to the

electron mean-free-path of Au (around ~40nm), there might be a coupling between the current fluctuations and the electrodes[5,9].

Supplementary note 6: Connection between a resonant antenna intensity enhancement $|K|^2$ and the Purcell factor F_p

We have presented an analysis of light emission by inelastic tunneling based on the point of view of fields radiated by current fluctuations. With that point of view, the electron to photon conversion efficiency can be enhanced by increasing the local field in the junction characterized by the enhancement factor *K*.

An alternative point of view can be used to deal with light emission by inelastic tunneling based on the Fermi golden rule. In that point of view, depicted in Fig. 1b, the electron decays by exciting either non-radiative modes or a gap plasmon mode. The contribution to the local density of states of the gap plasmon is thus the primary factor for the design of an efficient emitter. The efficiency appears as the result of a competition between the contribution to the density of states of the gap plasmon mode on one hand, the non-radiative modes on the other hand.

These two different pictures are not a priori equivalent. To establish a connection between these two visions, we briefly show that the *enhancement factor K* is proportional to the gap plasmon mode contribution to the radiative *local density of state*.

We start by considering an energy budget in stationary regime of a single antenna illuminated by a plane wave with incident field amplitude E^l_{inc}. By definition of the absorption cross section σ^l of the antenna, the absorbed power is given by

$$P^l = \frac{1}{2}\varepsilon_0 c\left|E^l_{inc}\right|^2 \sigma^l(\mathbf{u},\omega). \tag{26}$$

This absorbed power is equal to the power dissipated by the antenna mode which can be cast in the form:

$$P^l = \kappa V \varepsilon_0 \frac{\left|\mathbf{E}^l\right|^2}{2}, \tag{27}$$

where κ is the decay rate of the mode energy, V is the mode volume, and $\mathbf{E}$ is the electric field in the resonant antenna. By equating these two quantities, we derive the enhancement factor:

$$\left|K^l(\mathbf{u},\omega)\right|^2 = \frac{c\sigma^l(\mathbf{u},\omega)}{\kappa V}. \tag{28}$$

We now use the relation between the absorption cross-section and the gain $G^l(\mathbf{u},\omega)$ (not to be confused with the Green function introduced before) of the resonant antenna which characterizes the directivity of the emission angular pattern[12].

$$\sigma^l(\mathbf{u},\omega) = \frac{\lambda^2}{4\pi} G^l(\mathbf{u},\omega). \tag{29}$$

Inserting (29) into (28), and introducing the antenna quality factor $Q = \frac{\omega}{\kappa}$, we get

$$\left|K^l(\mathbf{u},\omega)\right|^2 = \left[\frac{3}{4\pi^2}\frac{\lambda^3}{V}Q\right]\frac{G^l(\mathbf{u},\omega)}{6} = F_p\frac{G^l(\mathbf{u},\omega)}{6}, \tag{30}$$

where F_p is the Purcell factor defined as the ratio of the local density of states in the antenna and the local density of states in vacuum.

Supplementary note 7: Antenna-based electroluminescence spectra when $k_B T \rightarrow 0$

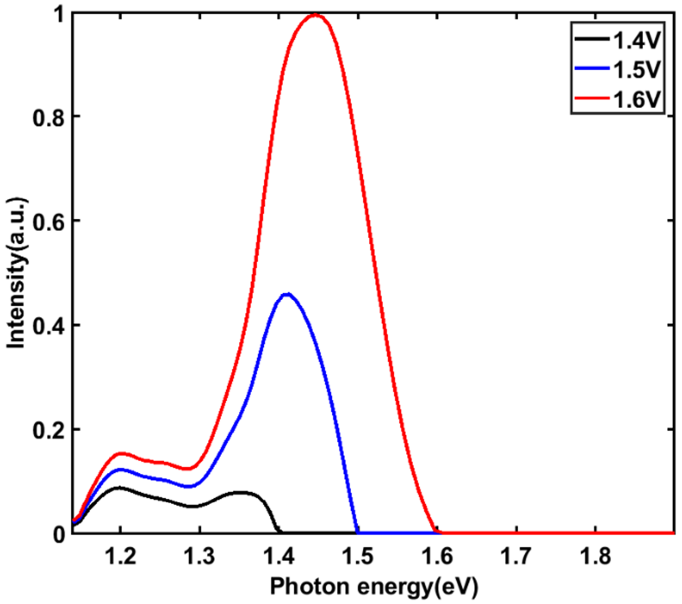

Supplementary Figure 9. Theoretical electroluminescence spectra (the same spectra as the figure 4(a-c) with $k_B T \rightarrow 0$) are shown with 3 different biased voltages from 1.4V-1.6V.

A key property of LEIT is a temperature-dependent cutoff close to *eV/h* due to energy conservation. Since our experiments are performed at ambient temperature, the Fermi-Dirac distribution is broadened by an amount on the order of a few $k_B T$. In particular, the cutoff at *eV/h* is smoothed. In addition, the resonance of the antenna enhances the emission beyond the cutoff resulting in a low visibility of the cutoff. We show in supplementary figure 9 the calculated form of the spectrum at 0K where it is seen that the cutoff at high energy depends on the applied voltage. We conclude that the lack of clear cutoff in our experiments results from the enhancement due to the antenna of the spectral tail of the current fluctuation spectrum due to the finite temperature.

Supplementary note 8: Tunable SPP emission spectra by antenna mode

It is well known that plasmonic patch antenna can be used to control the emission spectrum by simply modifying the parameters of the antenna[13,14]. We show in supplementary figure 10, that the emission spectrum varies when the width of the patch antenna varies from 130nm to 220nm as expected from a Fabry-Perot cavity. A redshift is observed for the second order gap mode. Here, we show the spectra without averaging over different widths. It is clearly seen that the spectra are narrower than the spectra measured experimentally. As we have considered a voltage at 1.6V, the spectrum vanishes at energies larger than 1.6eV.

When comparing the three resonant peaks, we observe an amplitude variation. Close to the cutoff *eV/h*, the amplitude decreases as expected. At lower frequencies, the emitted signal decreases because spontaneous emission rate decays and also because aluminium has larger losses.

Additionally, a small shoulder appears around 1.40eV. The position of the shoulder depends on the period of the antenna arrays so that we attribute it to a grating mode. Since the fabricated array of antennas is finite, this collective mode is too weak to be observed.

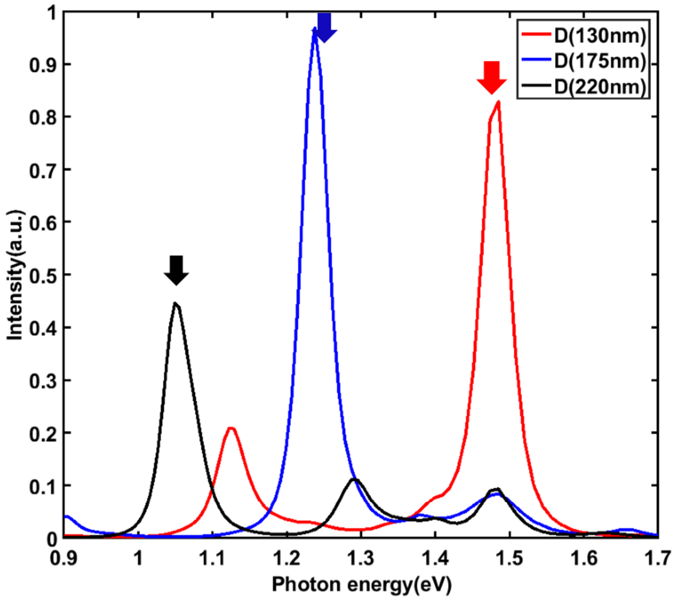

Supplementary Figure 10. Simulated spectra at voltage bias of 1.6V for the ASPEIT junction by varying patch width from 130nm, 175nm to 220nm. The vertical arrows show the resonance position of the second-order gap modes.

Supplementary note 9: Light emission by a planar junction.

In order to assess quantitatively the role of the antenna, we have measured the light emitted by a planar junction on the same sample with the same alumina barrier at a different position where no antennas were fabricated. Light emission by a bare junction formed by the intersection between an Au stripe (width 10 µm, thickness 50nm) and an Al stripe (width 100 µm, thickness 25nm), under a bias of 1.6V, is shown in supplementary figure 11. It is seen that close to the edges, emission is largely enhanced compared to the central area due to plasmon scattering. In order to extract photon emission only, we measured the power emitted in the intersection excluding the edges 20 µm from both sides, see the yellow dashed rectangle in supplementary figure 11.

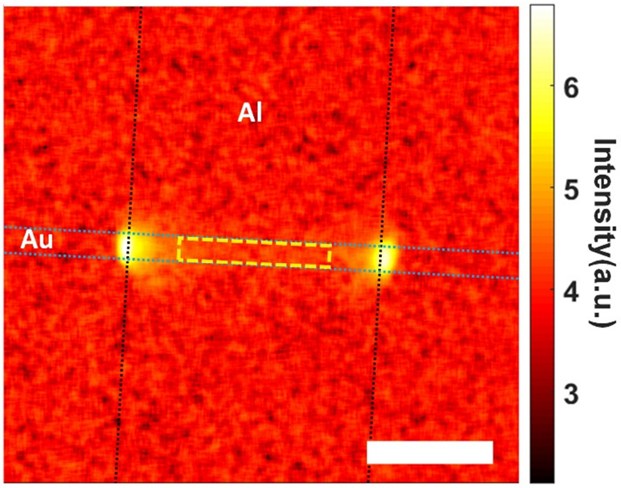

Supplementary Figure 11. Light emission from a macroscopic planar junction. Microscope optical image of a rectangular (10µm×100µm) planar junction at the crossing of an Al stripe and an Au stripe. Black and blue dashed lines are showing the edge of Al and Au electrode respectively. Scale bar is 50µm. Color bar is in natural log-scale.

Supplementary note 10: How to improve the electron to photon conversion efficiency

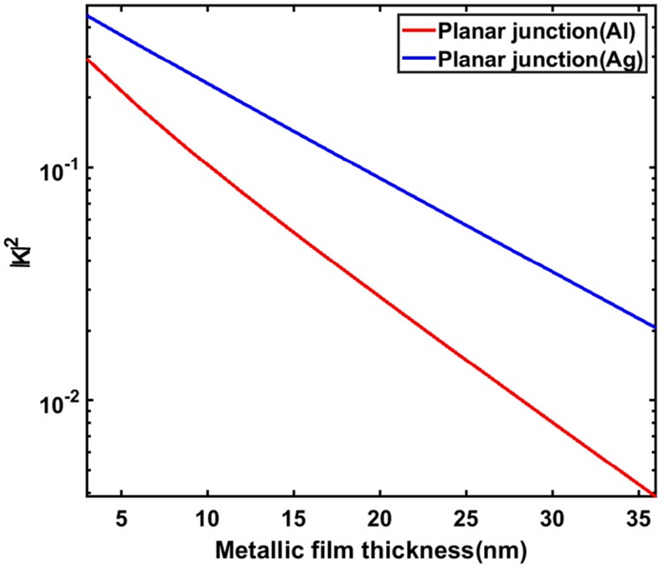

Supplementary Figure 12. Semi-log plot of $|K|^2$ (λ = 850nm), inside the barrier layer of the Al/AlOx(3nm)/Au(50nm) planar junction (red curve) and the Ag/AlOx(3nm)/Ag(50nm) planar junction (blue curve), are expressed as a function of the thickness of bottom electrode. Note the $|K|^2$ has been integrated over the collection solid angle.

As we have mentioned in the main text, the efficiency can be improved by tailoring the enhancement factor $|K|^2$. Here we discuss the impact of the bottom electrode thickness on this factor. The semi-log plot clearly shows the exponential dependence of $|K|^2$ as a function of the thickness. It is clearly seen that the electron to photon conversion efficiency decreases when increasing the bottom metal thickness. The relatively large thickness of aluminium explains the low photon output in our experiment. This plot shows that the plasmon to photon conversion decays exponentially with the metal thickness. This plot suggests that the electron to plasmon conversion is larger than the electron to photon conversion by at least one order of magnitude.

Replacing the electrode materials with a low-loss metal can reduce the intrinsic losses in the electrodes and increase the efficiency. We now investigate the photon emission of an antenna junction based on the Ag/AlOx/Ag configuration. We use the dielectric function of Ag from reference[15]. As shown in supplementary figure 13, the power emitted

on resonance integrated over the collection solid angle is enhanced by a factor of roughly 50 with respect to the Al-based antenna mode. The linewidth of the Ag-based junction presents nearly 1/3 of the Al-based junction.

We now estimate the electron to photon conversion efficiency with a silver antenna as follows. We compute theoretically the efficiency using either silver or aluminium and gold as in the experiment. Thus, we define an enhancement factor that is multiplied by the experimental efficiency. This procedure yields an estimated efficiency of 1.4×10^{-5}. We thus conclude that the electron to plasmon conversion should be larger than 10^{-4} for a silver antenna on silver substrate.

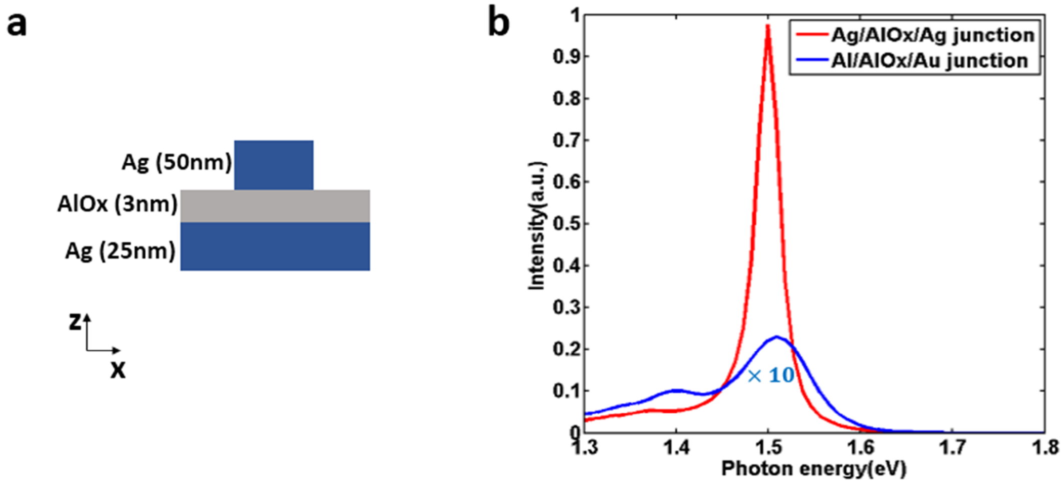

Supplementary Figure 13. (a) Schematic of the Ag/AlOx/Ag antenna junction. (b) The normalized photon emission spectrum between the Ag/AlOx/Ag junction and the Al/AlOx/Au with the same geometry (width=124nm) at a voltage bias of 1.6V. The spectrum of Al-based junction has been multiplied by a factor of 10. Note: the period of arrayed antenna is fixed at 400nm, and the dielectric function of Ag is taken from reference[15].

Supplementary note 11: Grating influence

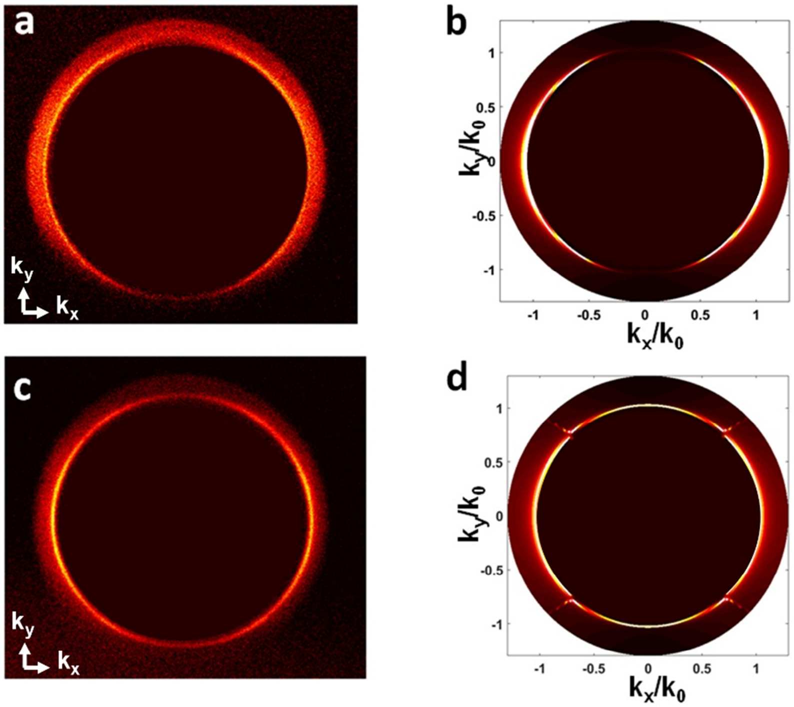

Supplementary Figure 14. **Back focal plane (BFP) images by varying the period of the antenna junction (same patch width as in figure 3).** (a-b) experimental and simulated BFP images with a period of 300nm, respectively. (c-d) experimental and simulated BFP images with a period of 600nm, respectively. Note: regarding the simulation images, we use a width of 120nm at wavelength of 850nm.

In this figure, we report the far-field emission pattern obtained when varying the antenna period. It is seen that the angular distribution is modified confirming that the angular structure of the emission pattern can be attributed to the periodicity of the antenna array.

Supplementary References

1 Lambe, J. & McCarthy, S. L. Light emission from inelastic electron tunneling. *Phys. Rev. Lett.* **37**, 923-925, (1976).

2 Kirtley, J., Theis, T. & Tsang, J. Light emission from tunnel junctions on gratings. *Phys. Rev. B* **24**, 5650-5663 (1981).

3 Février, P. & Gabelli, J. Tunneling time probed by quantum shot noise. *Nature Commun.* **9**, 4940, (2018).

4 Hone, D., Mühlschlegel, B. & Scalapino, D. Theory of light emission from small particle tunnel junctions. *Appl. Phys. Lett.* **33**, 203-204 (1978).

5 Laks, B. & Mills, D. Photon emission from slightly roughened tunnel junctions. *Phys. Rev. B* **20**, 4962-4980 (1979).

6 Rogovin, D. & Scalapino, D. Fluctuation phenomena in tunnel junctions. *Annals of Physics* **86**, 1-90 (1974).

7 Lee, H. & Levitov, L. Current fluctuations in a single tunnel junction. *Phys. Rev. B* **53**, 7383-7391 (1996).

8 Novotny, L. & Hecht, B. *Principles of nano-optics*. (2nd edition, Cambridge university press, 2012).

9 Kirtley, J., Theis, T., Tsang, J. & DiMaria, D. Hot-electron picture of light emission from tunnel junctions. *Phys. Rev. B* **27**, 4601-4611 (1983).

10 Davis, L. Theory of surface-plasmon excitation in metal-insulator-metal tunnel junctions. *Phys. Rev. B* **16**, 2482-2490 (1977).

11 Lalanne, P. & Silberstein, E. Fourier-modal methods applied to waveguide computational problems. *Opt. Lett.* **25**, 1092-1094 (2000).

12 Staelin, D. H., Morgenthaler, A. W. & Kong, J. A. *Electromagnetic waves*. (Pearson Education India, 1994).

13 Belacel, C. *et al.* Controlling spontaneous emission with plasmonic optical patch antennas. *Nano Lett.* **13**, 1516-1521, (2013).

14 Rose, A. *et al.* Control of radiative processes using tunable plasmonic nanopatch antennas. *Nano Lett.* **14**, 4797-4802, (2014).

15 Zeman, E. J. & Schatz, G. C. An accurate electromagnetic theory study of surface enhancement factors for silver, gold, copper, lithium, sodium, aluminum, gallium, indium, zinc, and cadmium. *J. Phys. Chem.* **91**, 634-643 (1987).
